# A Systematic Review of Event-Matching Methods for Complex Event Detection in Video Streams

**DOI:** 10.3390/s24227238

**Published:** 2024-11-13

**Authors:** Sepehr Honarparvar, Zahra Bagheri Ashena, Sara Saeedi, Steve Liang

**Affiliations:** 1 Department of Geomatics Engineering, University of Calgary, Calgary, AB T2N 1N4, Canada; zahra.bagheriashena@ucalgary.ca; 2 Department of Electrical and Software Engineering, University of Calgary, Calgary, AB T2N 1N4, Canada; ssaeedi@ucalgary.ca

**Keywords:** complex event detection, event processing, video processing, object detection in videos

## Abstract

Complex Event Detection (CED) in video streams involves numerous challenges such as object detection, tracking, spatio–temporal relationship identification, and event matching, which are often complicated by environmental variations, occlusions, and tracking losses. This systematic review presents an analysis of CED methods for video streams described in publications from 2012 to 2024, focusing on their effectiveness in addressing key challenges and identifying trends, research gaps, and future directions. A total of 92 studies were categorized into four main groups: training-based methods, object detection and spatio–temporal matching, multi-source solutions, and others. Each method’s strengths, limitations, and applicability are discussed, providing an in-depth evaluation of their capabilities to support real-time video analysis and live camera feed applications. This review highlights the increasing demand for advanced CED techniques in sectors like security, safety, and surveillance and outlines the key opportunities for future research in this evolving field.

## 1. Introduction

The increasing popularity of sensor systems, 5G networks, and the Internet of Things (IoT) has resulted in an abundance of online data streams and observations. Consequently, detecting meaningful events from IoT sensing systems has become a crucial task for various use cases [1]. Many of these events are complex since they are built by combining smaller events. Detection and identification of complex events support a better understanding of the environment and phenomena. Complex Event Detection (CED) supports scientists in detecting patterns of interest within undifferentiated data and events in real time [2]. Utilizing continuous processing of high-volume data streams, CED operates faster than traditional relational processing models [3]. It offers impressive capabilities for pattern finding and event matching in large event streams [4]. Additionally, CED addresses the challenges faced by data stream management systems (DSMSs) in managing complex patterns with sequential relationships [5]. Furthermore, it enhances the expressive subscription language of publish/subscribe systems, enabling the recognition and analysis of complex patterns in events [6]. Overall, CED and Complex Event Processing (CEP) are being developed for handling complex patterns and events in real time and for application in every use case which requires real-time complex pattern recognition.

In recent years, the consumption of video content has surged dramatically. According to a 2021 report by Coppola, people spent over 3.5 billion minutes (approximately 6654.5 years) watching videos in 2016, which skyrocketed to 12.2 billion minutes (around 23,195.5 years) by 2020. Meanwhile, during this period, the market for live camera feeds doubled [7]. Additionally, Market Research Future (MRFR) reported that the artificial intelligence (AI) camera market, valued at over USD 4.96 billion in 2018, is projected to exceed USD 21.58 billion by 2025 [8]. Leveraging AI capabilities, CED in videos can meet the growing demands for video content analysis and related services. CED in video streams is crucial for monitoring significant activities, such as repair tasks [9], identifying risky behaviors [10], and overseeing assembly operations [11]. Detecting complex events in unstructured data formats like videos involves recognizing specific spatio–temporal patterns within event streams. By utilizing camera feeds and computer vision, complex event processors can apply CED across various applications requiring visual event and environment perception [12].

CED in videos involves unique tasks such as object detection, attribute detection, and spatio–temporal relationship identification [13]. These tasks introduce distinct challenges, including frame delays, tracking losses, camera movements, environmental variations, and complex interactions between objects. Moreover, issues such as optimal camera positioning, lighting inconsistencies, pose variations, and background clutter need to be addressed. The primary challenge lies in achieving human-like event perception through consecutive video streams [14], necessitating the development of specialized spatio–temporal pattern modeling and event querying techniques. In summary, CED in videos can be distinguished from general CED in terms of data nature, event types, techniques, tools, and challenges.

Given these unique challenges, CED in videos warrants separate consideration from general CED. Many of these challenges revolve around event matching. Event matching in CED refers to the process of identifying patterns of events that satisfy certain predefined conditions or rules within a stream of events. This involves continuously monitoring event streams to detect occurrences of complex events, which are typically composed of multiple simpler events with specific temporal and logical relationships. Key aspects of event matching can be summarized as pattern specification, event stream processing, spatio–temporal constraints, and logical relationships. The main contributions of this paper are as follows:

- Reviewing the literature on complex events matching in videos over the past 12 years (2012–2024);
- Discussing how these studies have addressed gaps in CED in videos and outline considerations for future research in this field;
- Extracting the most critical challenges of complex event matching from these studies.

The remainder of the paper is structured as follows. The next section outlines a summary of the literature review method. Detailed information about CED definitions, its applications, and major challenges are elaborated in Section 3. Section 4 reviews complex event-matching methods from the past 12 years, in four categories. In Section 5, based on the literature review, we highlight the challenges of CED in videos. Section 6 discusses the popularity and trends of each event-matching method category. Finally, Section 7 presents conclusions and suggests directions for future research.

## 2. Research Method

**Figure 1:**
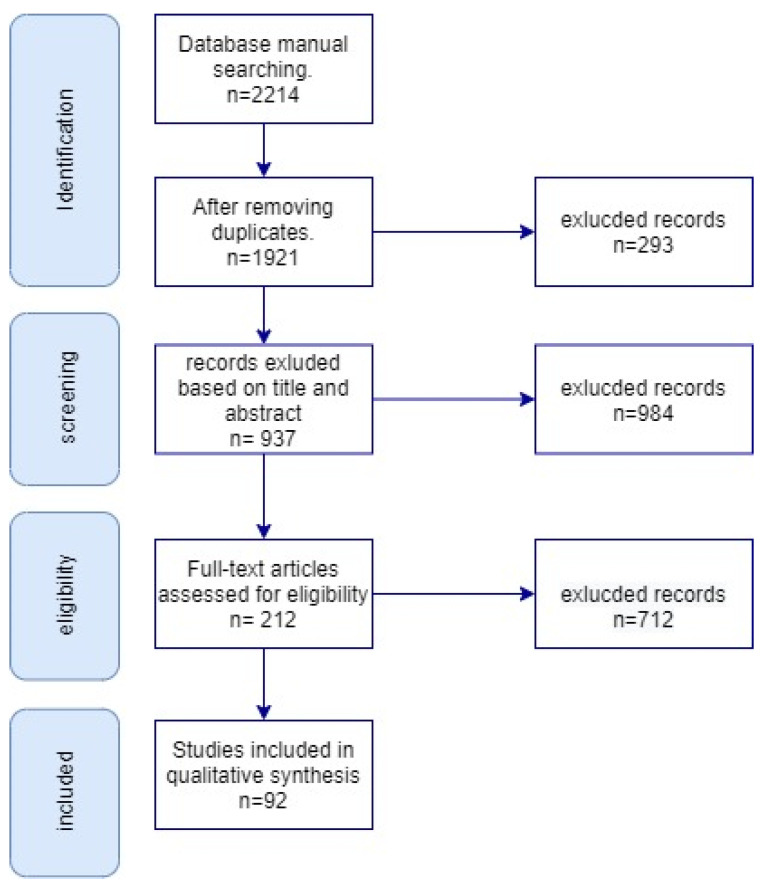
PRISMA flow diagram for the review.

To ensure a comprehensive and systematic review of the literature on complex event-matching methods in videos, we followed the PRISMA (Preferred Reporting Items for Systematic Reviews and Meta-Analyses) guidelines. We documented the selection process using a PRISMA flow diagram, outlining each step from the initial identification of records to the final inclusion of studies in the review. The flow diagram begins with the total number of records identified through database searches and manual searching. After removing duplicates, the remaining records were screened based on their titles and abstracts, leading to the exclusion of irrelevant studies. The full texts of the remaining articles were then assessed for eligibility based on predefined exclusion criteria. The exclusion criteria were studies published before 2012, industrial reports, languages except English, and studies without quantitative results. Studies that met the criteria were included in the final review. The flow diagram visually represents this process, showing the number of records at each stage and providing a transparent account of the decision-making process for study selection. Figure 1 presents the PRISMA flow diagram. In this diagram, the research record number for each step is indicated. The excluded records were studies filtered after each step. The final analysis in Section 6 is based on the included 92 records. The following describes the research items included in this systematic review.

### 2.1. Eligibility Criteria

We included peer-reviewed journal articles and conference papers published from 2012 to 2024 that were written in English and provided quantitative results relating to methods for complex event matching in video streams. Studies were excluded if they were published before 2012, were industrial reports, were not in English, or did not present quantitative results. We grouped studies based on the type of event-matching methods employed and their application contexts.

### 2.2. Information Sources

We searched the following databases and sources: IEEE Xplore and Google Scholar. The last search was conducted on 14 May 2024. We also performed backward and forward snowballing by reviewing reference lists of included studies and tracking citations of key articles to identify additional relevant studies.

### 2.3. Search Strategy

The search strategy involved using specific keywords and phrases such as “complex event detection”, “complex event process”, “event processing”, and “video object detection”. Boolean operators (AND, OR, NOT) and proximity operators were utilized to refine the search results.

### 2.4. Selection Process

A single reviewer screened the titles and abstracts of all retrieved records for eligibility. Full texts were assessed against predefined inclusion and exclusion criteria. The selection process is documented in the PRISMA flow diagram, which details the number of records identified, screened, and included in the review.

### 2.5. Data Collection Process

Data were extracted by a single reviewer using a standardized data extraction form. The form included study information, study design, methodology, outcomes, and general comments. No additional contact was made with study authors for clarification or missing data.

### 2.6. Data Items

We sought data on the following outcomes: accuracy of event matching, computational efficiency, and applicability to real-time analysis. We also collected data on participant characteristics and intervention details. Research with missing quantified results was noted and excluded from the studies for review.

### 2.7. Study Risk of Bias Assessment

We estimated the risk of reporting outcomes (e.g., qualitative vs. quantitative results), selection (e.g., the study poorly explained the matching algorithm), data input (e.g., real vs. simulated), and evaluation metrics biases (i.e., CED accuracy and performance). The risk items were categorized as low risk, high risk, and unclear risk. We scored high risk as 0.5, low risk as 1, and unclear risk as 0. The risk analysis demonstrated that 85 out of 92 studies had an average score of more than 0.5.

### 2.8. Synthesis Methods

We grouped the studies based on the type of event detection method used (e.g., object detection, spatio–temporal analysis, deep learning approaches) and compared the strengths, limitations, and applicability of each method. The challenges specific to each category were discussed, such as tracking issues, frame delays, and object occlusions. Finally, statistical analysis was conducted on the numbers of research studies in each category.

### 2.9. Reporting Bias Assessment

Certain categories, such as deep learning-based methods, might be more likely to be published due to the popularity of the topic, while less favorable or less “trendy” methods may be under-reported. To assess the bias, we examined publication trends and used a funnel plot to detect publication bias. Section 6 provides more details of the bias assessment results.

### 2.10. Certainty Assessment

To assess the certainty of the systematic review, we used the Grading of Recommendations Assessment, Development and Evaluation (GRADE) framework. We downgraded the certainty according to the risk of bias, inconsistency, indirectness, imprecision, and publication bias factors. Then, an overall uncertainty score was assigned to each category for each uncertainty factor. More details are explained in Section 6.

Table 1 represents the summary of the research method items and the assessment approaches.

## 3. Complex Event Detection

This section provides more context for authors about the definitions, applications, and famous commercial and open-source CED systems. We explain the general workflow of CED detection.

### 3.1. Definitions

Complex Event Detection is often defined as detecting composed events from timestamped simple or individual events [15]. Simple events are atomic events that do not include any sub-levels. For example, reporting the temperature at a certain time cannot be divided into sub-events [16]. Simple events are independent of other events and are also referred to as low-level events, which can be detected by sensors [17]. CED is handled by CEP engines [18] and can be considered as a sub-field of CEP [2]. CEP is the broader process that includes detection, analysis, and response to complex events. Complex Event Recognition (CER) refers to recognizing a composite event from simpler events to satisfy specific patterns [19]. These patterns capture the temporal and spatial relationships between simple events and facilitate the creation of meaningful complex events. CER leverages these patterns for real-time event detection, particularly in scenarios involving multiple events and data streams. In fact, CER focuses on the contextual interpretation and recognition of events. However, in some studies, CED, CER, and CEP are used interchangeably, and the distinctions between them are not clearly defined.

**Figure 2:**
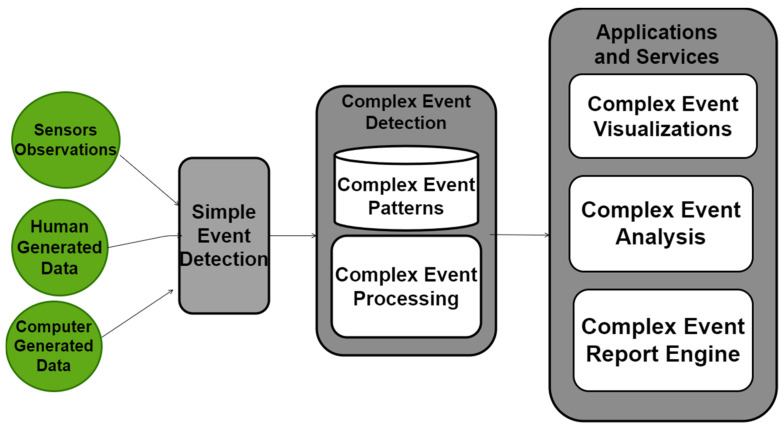
Complex Event Detection Workflow.

Figure 2 illustrates a high-level workflow for CED. This workflow is inspired by [20]. In CED, complex events are derived from diverse data sources, which can be classified as sensor data (e.g., RFID data) [21], human-generated data (e.g., social networks content) [22], or computer-generated data (e.g., bank transactions) [23]. These data streams are then processed to identify simple events, which can be detected based on straightforward conditions, such as surpassing a threshold within a specific time and location. Fundamentally, detecting simple events involves identifying significant occurrences within the data [24]. The detected simple events are subsequently fed into the CED process as event streams, using CEP engines to identify complex events. These complex event streams can be utilized in various applications and services for visualization purposes [25], analytics [26], or reporting [27]. For each service, there is a complex event patterns database that stores predefined rules for constituting complex events from simple events.

### 3.2. Applications and Frameworks

Detection and processing of complex events has been widely applied in different applications such as health monitoring [28], fraud detection [29], financial services [30], fault-tolerant systems [31], smart hospitals [32], etc. One of the interesting applications of CED is health monitoring. For example, CED can be used to detect when the hands of patients entering the room are not sanitized [33]. Upon detecting a risky action in an event-driven architecture, a warning is sent to healthcare workers [34]. CED can also be used in human activity pattern detection. Smartphone devices can be used to collect users’ routine activities. A CEP engine can detect the relationships between frequent activity patterns and find the activity patterns of users [35]. CED finds an intriguing application in handling massive complex state and event analysis on Internet of Things (IoT) networks. For instance, CED can efficiently detect traffic congestion by processing vast event streams from GPS devices [36]. Furthermore, CED proves useful in managing sensor maintenance in IoT environments, assessing the need for replacement or repair based on observations and the behavior of the sensors themselves [37].

Due to the high demand for complex event applications, many CED architectures, systems, and software programs have been produced, such as KafkaStreamsCEP (1.0.0) [38], Eagle (1.0.0) [39], Rivus-CEP (1.0.0) [40], and GoStream (1.0.0) [41]. One of the most famous services and APIs for CED is FlinkCEP, which has been developed by the Apache Software Foundation and is supported by the Apache Flink framework. It provides powerful parallel processing to resolve expensive computer cluster solutions for CED [42]. Esper [43] is another well-known CED engine that works like an upside-down traditional relational database, building relations based on users’ queries and data streams [44]. Esper uses and compiles Event Processing Language (EPL) into bytes to manage event matching. EPL is a declarative language to support data streams and event streams, which uses a lot of Structured Query Language (SQL) functions and commands [45]. Siddhi’s [46] platform provides microservices and an event query API for streaming and CEP. It offers the Siddhi query language for CED querying. Built upon the Siddhi core, the WSO2 server supports a wide range of immutable and mutable data. Table 2 provides a summary of CED frameworks.

### 3.3. Complex Event Frameworks Challenges

Most of the well-known software on the market focuses on improving the performance of CEP. However, greater challenges are present, such as big data processing, semantic modeling, and managing uncertainty. One of the challenges of CED is the semantic modeling of event relationships to build complex event patterns [47]. Some of the existing solutions for modeling semantics in CEP have proposed semantic query languages for event streams, such as C-SPARQL [48] and EP-SPARQL [49]. Other approaches have proposed ontology formalization and rule-defining enhancement [50,51,52]. Performance remains one of the most critical challenges in CEP. The event detection metric is widely used as the primary indicator of CEP performance, and significant research has focused on reducing it [53,54]. Moreover, big data processing presents a critical challenge particularly when dealing with vast event streams from various sources in networks like the IoT. Apache Storm [55], SCEPter [56], and CEP-based pub/sub [57] are considered potential solutions for big data in CED. Finally, uncertainty is one of the fundamental issues in CED. Handling CED uncertainty involves various parameters such as time uncertainty, attribute and model uncertainty, computational delay, or online inference. The solutions for the uncertainty issue are based on complex event estimation likelihood using probabilistic or stochastic models [58,59,60] and provide improved detection efficiency [17,61,62]. In the next section, we focus on studies aimed at improving event matching in videos involving uncertainty and semantic modeling challenges.

## 4. Event-Matching Methods

The primary objective of this section is to present a comprehensive review of various complex event-matching methods. In our review, we used a correlation matrix to evaluate the distinctiveness and overlap among different methods used for complex event matching in video streams. By examining intersections in methodological features, we were able to determine the degree of alignment or overlap among categories, specifically analyzing the presence of shared techniques across groups. The matrix highlighted limited but notable overlaps, particularly between multi-source solutions and other categories, confirming that these methods have unique integration-focused characteristics with minimal intersection. Similarly, minor overlaps between object detection and spatio–temporal matching and approaches based on training and predicting suggested that while neural networks were used in both, they served different roles across categories. Based on this analysis, we concluded that the four categories—object detection and spatio–temporal matching, approaches based on training and predicting of videos, multi-source solutions, and others—provided the best categorization, capturing distinct methodological approaches while acknowledging limited overlaps that further validated the robustness of our classification scheme. The methods discussed in this review have been categorized into the following sub-sections, each of which includes examples of relevant studies in their respective categories.

### 4.1. Multi-Source Solutions

Detection of complex events in video streams often necessitates the integration of supplementary data sources to improve accuracy and robustness. An effective strategy involves incorporating audio observations alongside visual information, thereby increasing flexibility in event matching. For instance, Natarajan et al. applied feature extraction techniques across video text, audio, and video frames, utilizing kernel-based feature fusion to discern complex events within video streams [63]. Similarly, Jhuo et al. sought to enhance the performance of bimodal (audio/video) CED by constructing a bipartite graph that encapsulated relationships between Bag-of-Words (BOW) representations of audio and video content [64]. This approach facilitated a more comprehensive understanding of event contexts. Oh et al. emphasized the significance of integrating audio data in CED, highlighting its potential to augment the accuracy of low-level video features, thereby enabling a hierarchical classification of events from basic to advanced levels [65].

In a parallel endeavor, Shahad et al. proposed an intelligent surveillance system that synergistically utilized data from door sensors and cameras [66]. By treating camera and door sensor observations as low-level events and aggregating them within temporal windows, the system could generate alerts for complex events based on predefined rules. Moreover, Honarparvar et al. addressed the challenge of object tracking loss in videos by leveraging an IoT-based framework [34]. This framework facilitated the establishment of spatial and temporal relationships among objects captured by disparate cameras. By unifying object data into a common spatial reference system, the framework mitigated the risk of losing object continuity across camera views.

In a more recent development, Brousmiche et al. introduced the Multimodal Attentive Fusion Network (MAFnet) architecture, designed to elevate event recognition accuracy through a nuanced fusion of audio and visual cues [67]. This architecture divided videos into discrete time windows (clips), from which both audio and video features were extracted. Subsequently, temporal and multimodal attention modules were employed to assign importance scores to the video clips based on their contribution to complex event formation. The integration of a feature-wise linear modulation (FiLM) layer served to harmonize these modules, ultimately determining the occurrence of complex events with heightened precision and efficiency. The audio and video also could be concatenated in a simple natural language processing (NLP) neural network to predict complex events, able to provide high accuracy and performance [68]. Gao et al. proposed an audio–video representation learning (AVRL) framework to fuse audio and video prediction in a supervised simple NLP. They used a 3DResNet convolutional neural network (CNN) to predict events in videos and predicted audio events with a VGG CNN. The results were then classified into event categories based on the audio and video prediction results.

### 4.2. Approaches Based on Training and Predicting of Videos

**Figure 3:**
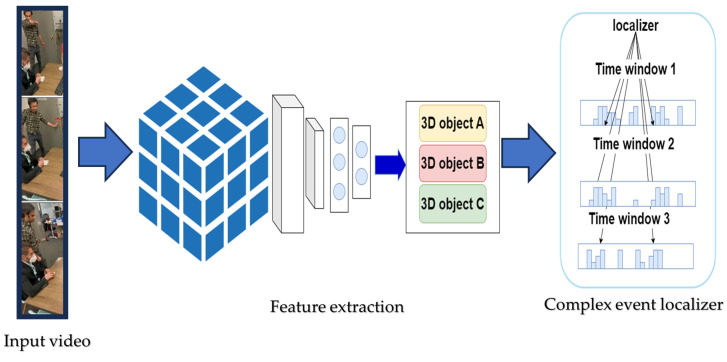
Workflow of training and prediction approach for CED.

The other major approach involves training supervised or semi-supervised models for a set of labeled videos and using them to predict complex events in new videos. Figure 3 represents the general steps for approaches based on the training and prediction of videos. In this method, a video is split into a certain set of time windows. The split videos are processed by supervised machine learning to extract spatio–temporal features. In the final step, the features are localized using the localizer algorithm for each time window, and complex events are identified in a certain timestamp and location. Ma et al. leveraged video-level attributes as a set of videos including basic actions to fix the issue of characterizing dynamic properties at the image level [69]. They proposed multi-level collaborative regression (MCR) using attributes as cues for the detection of complex events. The latency problem of event recognition in videos was addressed by training the CED model based on duration, transition, and appearance parameters [70]. Another noteworthy contribution is the utilization of adaptive relatedness analysis to establish matches between labeled video frames and new videos. This approach enables effective linkage between labeled data and novel video inputs. It also enhances the event recognition system’s performance and adaptability [71].

Feature extraction is one of the most important components of CED. In some cases, feature extraction might lead to the loss of temporally local information when quantified information is pooled from a global vector. Therefore, a video could be split into instances (i.e., a subset of the video with a certain length) formed by pooling local features into segment levels. Then, instances are classified using multiple instances (MIL) and a support vector machine (SVM)-like algorithm [72]. One of the issues of this approach is the ambiguity of exemplars when familiar words are included. For example, “changing laptop keyboard” has words in common with “changing keyboard language”, while they infer two different actions. Xu et al. addressed this issue by proposing a cross-feature reasoning approach to assign optimal labels to each complex event [73].

In 2015, DevNet was revealed as a deeper CNN that leveraged spatial and temporal key evidence in video frames to detect complex events [74]. In other words, it applied fine-tuning image features on key spatial–temporal events in frames. DevNet was developed to detect high-level complex events and localize this key evidence. The unavailability of labeled videos poses another significant challenge in training CNN for videos. A combination of improved dense trajectories (IDTs) and extracted semantic features enabled CED to classify events even without labeled events in videos. Abbasnejad et al. used IDT to extract temporal features and a deep convolutional neural network (DCNN) to extract semantic features [75].

One of the problems of event matching is the negative effect of fusing multiple features in the CED process. Liu et al. used a primal SVM which provided approximate prediction with a gradient descent method [76]. The transformation of time-series images into cells provided the opportunity to analyze multiple factors on a cell-by-cell basis, enabling the detection of event patterns. This approach allowed a more fine-grained and comprehensive examination of data, leading to improved event-recognition capabilities. Dao and Zettsu attempted to detect car accidents as complex events by analyzing traffic and rainfall satellite images within certain geofences in a certain time window [77]. They created a video by multiplying image geofences and sorting them in time. Then, they trained a 3D deep CNN model to predict accidents based on geofence maps and an SVM classifier to detect the complex events in time.

Xu et al. introduced Single-StreamLine (S2L) to solve the failures in optical flows when extracting features at the video level [78]. Arif et al. addressed the optical flow low accuracy issue by designing a modality-based CED framework that only uses raw RGB frames as input. The proposed framework extracts semantic (i.e., rotation and lo-cation invariance) and spatial features (i.e., shape and geometry) into two maps. Then using a pixel-wise fusion and attention-based cleaning, a final feature map is produced for each frame. In the last step, all frames are represented in vectors by Long Short-Term Memory (LSTM) encoders to represent the complex events at the video level. One of the issues of CED is the variety of low-level features quality in the videos. Lou et al. proposed an algorithm, inspired by curriculum learning, to train the classifier with high-quality instances and gradually add lower-quality instances to get optimum training [79]. One of the challenges with existing CED approaches was their restriction to a fixed number of frames when training CNNs on time series data. To address this limitation, Alanzi and Muhammad introduced a 4-stream (or branch) architecture known as 4S-3DCNN. This architecture merges every four frames and emphasizes both spatial and temporal information in the combined frames, facilitating the im-proved matching of event patterns [80]. Vilamala et al. (2023) proposed DeepProbCEP as a ProbLog approach to enable training complex events with smaller datasets. Unlike traditional approaches, DeepProbCEP does not use a neural network for complex events reasoning. It takes advantage of DeepProblog as a probabilistic logic coding ap-proach to let users modify the complex events’ rules and eliminate CNN false results [81]. In a most recent research, Fei et al., have proposed SumNet as the combination of object CNN and scene CNN to increase the performance of summarizing videos into meaningful complex events. For each event, a linear weighted function is defined which includes the prediction scores of objects and scenes prediction [82].

### 4.3. Object Detection and Spatio–Temporal Matching

**Figure 4:**
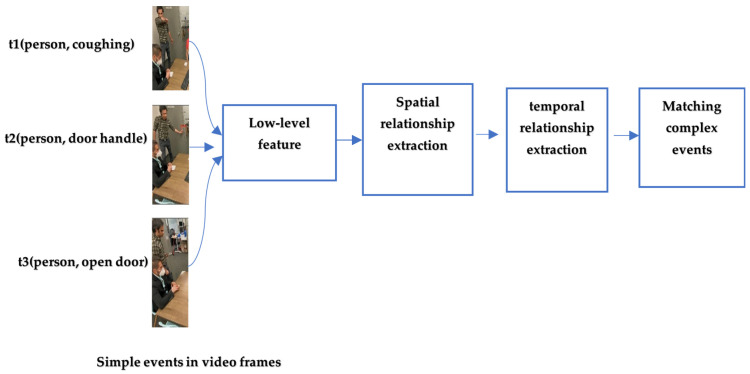
Object detection and spatio–temporal matching general workflow.

Figure 4 illustrates the general workflow for approaches based on Object detec-tion and Spatio-temporal matching. Videos are composed of frames that contain nu-merous objects, which can be detected by deep-learning object detection models. Ex-tracting these objects enables the capture of spatial and temporal relationships within a frame and across consecutive frames. Uncovering these relationships plays a crucial role in aligning predefined patterns with the detected events, enhancing the overall CED process. Entity-centric feature pooling is an example that uses object detection and spatiotemporal relationship extraction for event matching [83]. This approach was specifically proposed to find events based on objects’ interactions with humans. Therefore, an area of Interest for every frame is mapped based on detected objects in the human actionable area and human gaze. Events are then pooled using localized spatial pooling.

The EventNet is one of the pioneers of applying object relationship matching in CED. This framework introduced the concept of a complex event ontology within videos [84]. Additionally, it proposed a novel approach to associate events with concepts, facilitating the querying of events within video content. This innovative framework marks a significant advancement in effectively representing and querying complex events in video contexts. Not to mention that EventNet supports human-related events by providing a hierarchical ontology of events from high levels to involved objects in lower levels. Complex events can be recognized by analyzing moving object trajectories. Ke et al. proposed a framework to pair labels and trajectory vertices in a hypergraph to assign semantic concepts to visual events in video streams [85,86]. Cosar et al. developed a unified framework for abnormal behavior detection of humans in videos [87]. They tracked objects and formed trajectories in frames using pixel-wise descriptors. Then, they extracted dense trajectories using grid-based analysis and snapping trajectories. These trajectories are clustered based on location, speed, direction, and bounding box descriptors to detect abnormal behaviors. Li et al. split events into a hierarchy [88]. In the lowest level of the hierarchy, frame features are extracted, and static visual concepts are built upon the features. In this hierarchy, temporal relationships between the static concepts create activity concepts. They claimed that the error propagation problem effect on CED is significantly reduced by the proposed approach.

Querying events in this complicated structure, such as a hierarchy of features is challenging. Therefore, some studies tried to improve the querying performance in complex event matching. VideoStorm improved the quality of video queries by lag and quality-aware querying [89]. It presented an architecture that comprises a central manager and worker machines. This design enables the intelligent distribution of resources, ensuring optimal allocation for each specific video query. Kang et al. addressed the computational challenge of querying videos by introducing BLAZEIT [90]. They proposed FrameQL as a tool that empowers users to execute video analytics queries and create optimized query plans aimed at reducing calls to expensive DNNs. Moreover, Khan et al. proposed a two-phase workflow including the simple event detection pipeline and the complex event detection pipeline [91]. In the first phase, objects within videos are detected and tracked. Subsequently, employing event calculus and logical reasoning, the system identifies the occurrence of simple events at specific time points as indicators of complex events.

Some scholars have attempted to improve event models in complex event matching by incorporating more realistic semantics of event relationships and enhancing query event languages to support more complicated events. One significant advancement in this area is VidCEP, proposed by Yadav and Curry as a real-time video CEP framework. VidCEP leverages the Video Event Query Language (VEQL) and an event-matching engine to detect complex events. The authors argued that existing video CEP systems only support fixed data models and temporal event matching, whereas VEQL enables VidCEP to match events over spatial and temporal patterns using various data models [13]. Additionally, Yadav and Curry introduced the Video Event Knowledge Graph (VEKG) to model video objects and spatial-temporal relationships as complex event patterns [92]. In this model, entity nodes represent video objects, each including various attributes, and edges represent intraframe (spatial) and interframe (temporal) relationships. To model spatial relationships, the framework employs geometric, topology-based, and direction-based relationships. Temporal relationships are modeled using Allen intervals. VEKG and VEQL together facilitate a multistep complex event-matching procedure [93]. Within the suggested matching method, a window assigner allocates windows to video streams according to specified query parameters. A state manager then receives the window states (i.e., the most recent batch streams in the windows) and sends them to a matcher. Finally, VEKG of complex events are converted to graphs and matched to the graph represented by the user using VEQL.

To address the modeling of semantic relationships between objects in video streams, Yadav and Curry developed the Multimedia Event Relation Network (MERN). MERN supports complex event pattern modeling in five layers: the event layer, detection layer, semantic knowledge layer, event pattern rule layer, and event calculus layer. It connects the query layer to an external knowledge base, providing pattern ontology models for the event matcher [94]. To reduce the complexity and processing time for CED, Yadav et al. introduced the VEKG - Time Aggregated Graph (TAG). This approach decreases the number of nodes in complex event pattern graphs by including an additional temporal dimension to represent the graph at different timestamps, thus simplifying the event-matching process [95]. Furthermore, Ashish et al. introduced an NLP-guided ontology to query visual information. This approach addresses the issue of expensive manual ontology design by introducing a semi-automatic NLP-guided method [96]. In the most recent development, Kossoski et al proposed the Notification Oriented Paradigm (NOP) aimed at reducing the latency of event matching. NOP simplifies event matching into Static Query Chain (SQC) and Dynamic Query Chain (DQC), which reduces storage, retrieval, and trigger times. However, while SQC and DQC simplify many complex spatial and temporal relationships, NOP remains limited to specific types of complex events [97]. These advancements reflect the ongoing efforts to enhance complex event matching by improving the semantic modeling of events and optimizing the efficiency of event detection frameworks.

### 4.4. Other Solutions

Some solutions to CED in videos diverge from traditional categorization. These include methods such as complex event matching using descriptors, unsupervised methods, zero-exemplar techniques, semantic search algorithms, and keyword extraction. Alt-hough research in these areas is not yet extensive enough to establish new categories, these methods still present valuable alternative approaches for addressing complex event-matching issues. For instance, complex events can be extracted from videos us-ing feature extraction methods. These features can be static (e.g., Gini-Index Text (GITS), Scale-Invariant Feature Transform (SIFT), and colorSIFT) or dynamic (e.g., Mo-tion SIFT (MoSIFT), Spatio-Temporal Interest Point, and Spatio-Temporal Interest Point). Once extracted, these features can be classified into different events using BOW descriptors and unsupervised methods [98]. Developing efficient concept-matching algorithms can significantly enhance CED performance.

Yan et al. introduced a framework designed to rapidly identify high-level events by establishing similarities between event-kit descriptions and the Semantic Index da-taset (SIN), which comprises visual concepts [99]. The zero-exemplar framework emerged as a solution to identify complex events without any visual examples [100]. This framework proposed a semantic search algorithm to match user-generated textu-al queries with video corpus information, improving video search and training proce-dures. De Boer et al. demonstrated that query expansion could enhance the perfor-mance of zero-example methods by adding more words to queries [101]. Furthermore, Li et al. improved zero-shot CED through a three-step workflow involving semantic search, concept selection, and Event-Adaptive Concept Integration (EACI). Semantic search measures the semantic correlation between concepts and query events using NLP, ranks these concepts, and selects the top ones for each event. By combining EACI with the Area Score Under Curve (ASUC), they determined the closest-matching con-cept for each event [102]. 

Building on these approaches, Li et al. proposed a ranking-based method to match visual frames with concepts, thereby improving zero-shot CED accuracy [103]. They used keyword extraction and word vectors to find semantic concepts from textual in-formation instead of relying solely on event names. Traditional zero-shot CED focuses on first-order concepts like scenes, objects, and visual relationships, which are often overlooked. To address this issue, Jin et al. developed a library of high-order concepts such as visual relationships and complex human actions [104]. They used NLP to ex-tract triplets from image captions—comprising subject, predicate, and object—as rela-tionships in each image. Spectral clustering was then applied to remove noise and re-dundancies from these relationship sets, preparing them for concept training.

Table 3 demonstrates a summary of advantages and disadvantages of event-matching methods.

## 5. Challenges of Complex Event Matching

Based on our comprehensive review of the literature, we categorized the challenges associated with complex event matching into several key areas. These challenges are derived from the limitations and future works of the studies considered in Section 4. Additionally, we evaluated each category of methods—multi-source solutions, event matching by training and predicting, object detection and spatio–temporal matching, and other solutions—on their ability to address these challenges. Using a composite metric developed from five ranking criteria—accuracy, robustness, low latency, scalability, and ease of implementation—we provide intra- and inter-category rankings to guide future research efforts.

### 5.1. Composite Metric for Method Evaluation

The composite metric evaluates each method across the following criteria:

- Accuracy: How effectively the method detects and matches events.
- Robustness: The ability of the method to handle noisy or incomplete data.
- Low Latency: Efficiency of the method in real-time applications.
- Scalability: How well the method adapts to larger datasets or complex events.
- Ease of Implementation: Practicality in terms of computational resources and required expertise.

Each method is scored based on these criteria, and a composite rank is generated to evaluate their overall effectiveness in complex event detection (CED).

### 5.2. Intra-Category Ranking

Table 4 provides the detailed intra-category rankings for the event-matching methods.

- **Multi-source Solutions**
  1. **IoT Framework:** The IoT framework ranks highly across accuracy, robustness, scalability, and latency, although it scores lower on ease of implementation. Its strengths lie in integrating data from multiple sensors, which enhances accuracy and robustness while allowing for a moderate reduction in latency.
  2. **Multimodal Feature Fusion:** This method scores high in accuracy but falls short in scalability and ease of implementation. It ranks moderately due to its balanced performance across most criteria but lacks consistency in latency optimization.
  3. **Low-Level Feature Integration and Hierarchical Event Fusion:** Although this method ranks well in accuracy, its limitations in robustness and scalability lead to a lower overall ranking.
- **Event Matching by Training and Predicting**
  1. **Merging Frames and Vector Encoders:** This method ranks highly across accuracy, latency, and moderately in robustness. It is particularly effective in scenarios where timely event matching is critical.
  2. **Multiple Instances Learning:** MIL ranks well in accuracy and robustness but lacks in scalability and ease of implementation. It is a strong candidate for environments where robustness is prioritized.
  3. **Spatial and Temporal Key Evidence:** This approach shows consistent performance across all criteria, making it well-rounded but without excelling in any single dimension.
  4. **Cell-by-Cell Basis Feature Fusion:** Despite high accuracy and robustness, this method’s significant challenges with latency and scalability limit its overall ranking.
- **Object Detection and Spatio–temporal Matching**
  1. **Event Knowledge Graph and Dividing Objects into Static and Dynamic Categories:** This method excels in accuracy, robustness, and scalability, making it ideal for scenarios requiring high accuracy and adaptable event matching.
  2. **Event Query Optimization:** With high rankings in low latency and scalability, this method performs well in real-time applications where speed is prioritized over accuracy.
- **Other Solutions**
  1. **Zero Examplers and NLP-Aided Feature Extraction:** This approach performs highly across robustness, scalability, and ease of implementation, making it valuable for environments where innovative feature extraction is needed.
  2. **Feature Extraction and Bag-of-Words (BOW) Descriptors:** With moderate scores across all criteria, this method is balanced but lacks standout strengths.

### 5.3. Inter-Category Ranking

While each category serves a unique domain of application, an inter-category ranking offers insight into the overall effectiveness of methods:

- **Top Performers**

*IoT Framework (Multi-source Solutions)* and *Event Knowledge Graph (Object Detection and Spatio–temporal Matching)* rank highest across multiple criteria, making them the preferred choices for achieving both high accuracy and robustness in complex event detection. The *Zero Examplers and NLP-Aided Feature Extraction* (other solutions) method is also ranked highly due to its ability to address challenges in robustness and scalability effectively.

- **Balanced Approaches**

*Multiple Instances Learning (Event Matching by Training and Predicting)* and *Event Query Optimization (Object Detection and Spatio-temporal Matching)* both score moderately well across a range of criteria. They are ideal for environments that require balanced performance in both speed and accuracy without extreme optimization in either direction.

### 5.4. Event-Matching Challenges

In this subsection, we review challenges of event matching in videos, extracted from the future works or limitations of the studies in Section 4. The challenges are as follows:

- **Object Detection and Tracking**

Losing object tracking due to poor image detection accuracy and false negatives poses a significant challenge to spatio–temporal pattern matching of complex events. Due to the hierarchical nature of complex event modeling, inaccuracies in detecting objects can lead to false negatives for Complex Event Detection (CED). “*Object Detection and Spatio-temporal Matching*” methods rank highly in addressing this challenge due to their enhanced object detection accuracy and robust tracking mechanisms, making them particularly effective at mitigating the effects of lost tracks. Methods like the *Event Knowledge Graph* and *IoT Framework* rank highly due to their enhanced detection accuracy and ability to maintain object tracking.

- **Small Training Dataset Size for Visual Examples**

The limited size of labeled datasets poses a challenge in video search applications. The majority of research within the “*Event Matching by Training and Predicting*” and “*Other Solutions*” category has recognized and addressed this issue by exploring approaches like zero-shot feature extraction, which ranks highly in handling small training datasets by using semantic information to compensate for limited data availability. This method ranks highly in addressing this challenge by leveraging NLP techniques for effective feature extraction without large amounts of training data.

- **Optical Flow Failure**

Optical flow failure, often caused by irregular camera movements or large-scale perspective changes, can generate false negatives in video CED. “*Object Detection and Spatio–temporal Matching*” approaches are designed to overcome these challenges by employing more sophisticated motion analysis techniques, making them better suited to address failures in optical flow.

- **Noisy and Unreliable Labels**

Noisy and unreliable labels are a critical problem for the training approach in CED. Labels are often autogenerated and assigned to groups of instances rather than individually, resulting in low precision in training CED classifiers. The “*Event Matching by Training and Predicting*” and “*Other Solutions*” categories rank highly for addressing this challenge by leveraging advanced training methodologies to improve the quality of labels generated.

- **Latency Problems in Event Recognition**

The latency of CED is a critical issue, especially in online and real-time applications, as low event-matching speeds can lead to failures in tracking or mapping trajectories. The “*Multi-source Solutions*” category ranks moderately here, as integrating multiple data sources helps optimize event matching and reduce latency. However, these methods require further development to fully address real-time latency challenges. The IoT framework and Event Query Optimization methods both score well for their ability to reduce latency, making them suitable for real-time CED applications.

- **Designing Ontology of Objects and Relationships for Complex Events**

The construction of ontologies is vital for CED as it facilitates the extraction of spatial and temporal relationships in videos. This challenge is largely addressed by the “*Object Detection and Spatio-temporal Matching*” category, which ranks moderately for exploring automated ontology generation, albeit with limitations that require further advancements in automating the process. The Event Knowledge Graph ranks highly in addressing ontology construction for CED, leveraging its ability to categorize objects effectively and extract meaningful relationships.

- **Spatial Relationships Extraction**

Occlusion and complex spatial relationships present significant challenges for extracting spatial data from videos. Methods within the “*Object Detection and Spatio–temporal Matching*” category rank highly in handling occlusion and accurately extracting spatial relationships using geometric and topological analysis techniques, making them effective for this particular challenge.

Table 5 includes a summary of event-matching challenges and related intra-category top ranked solutions.

## 6. Discussion

**Figure 5:**
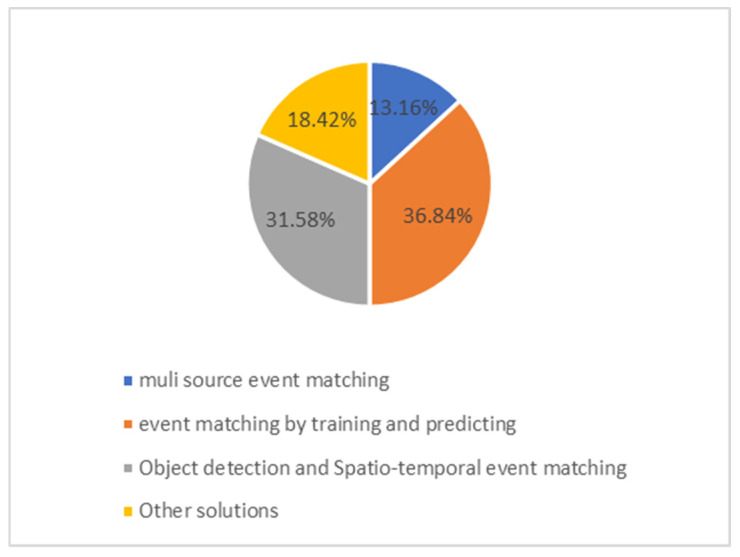
Portions of published event-matching solutions in 12 years (2012–2024).

In this section, we discuss research studies focused on event matching and rea-soning for CED that have been published since 2012. Initially, we summarize the num-ber of studies according to the categories outlined in Section 4. Figure 5 illustrates the portion of the total number of published papers in each category. According to the da-ta presented in Figure 5, the majority of the proposed solutions fall under “Event matching by training and prediction”. These solutions are mostly applied to detect complex events in YouTube videos when some textual information such as descrip-tions or subtitles is available. These methods which attract the most attention in aca-demia enable smarter video content search on the web. Such methods, which have garnered significant attention in academic research, facilitate smarter web-based vid-eo content searches. “Object detection and Spatio-temporal event matching” solutions are more frequently applied for surveillance and real-time environmental monitoring cases. Using multiple sources to improve CED event matching is the least popular solu-tion for CED in the last 12 years. However, integrating observations from various sources to process complex events recently has been applied in several applications such as precise agriculture [105], transportation [36], proactive business management [106], etc. Due to the advancement of multisource CED in other fields, there is an anticipated increase in publications related to multisource CED in video contexts in the future.

### 6.1. Research Trends

**Figure 6:**
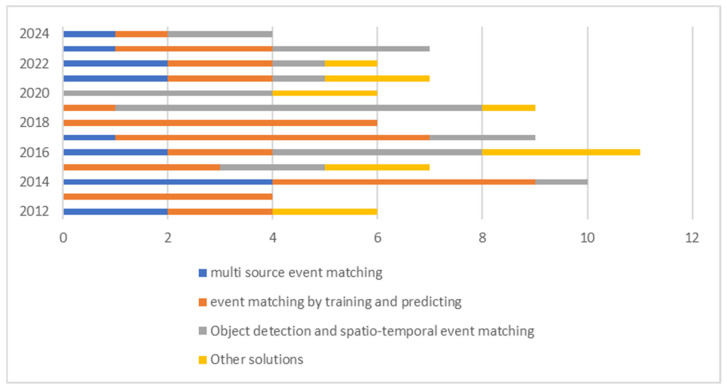
Published event-matching solutions by year (2012–2024).

Figure 6 shows the number of publications in each category in each year. Among solutions, “Object detection and spatio–temporal event matching” has shown the most growth in recent years while “Event matching by training and predicting” has attracted less attention in the past three years. Multisource solutions in 2021 received the most publications in the last three years of the study period. The number of publications dedicated to innovative solutions in CED that do not fit into any predefined category has remained consistent over the past decade.

**Figure 7:**
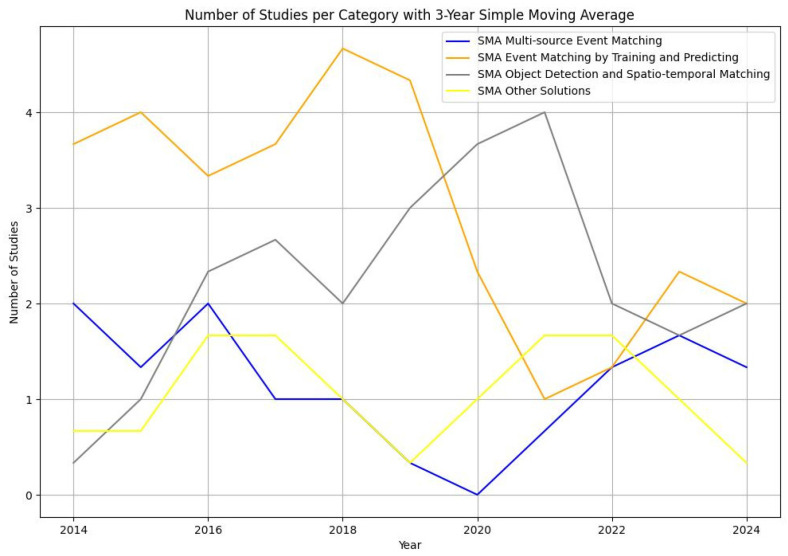
Trends of method categories by 3-year SMA.

To further analyze category trends and predict future research directions, we calculated a 3-year simple moving average (SMA) for each category (Figure 7). The SMA helped smooth out short-term fluctuations, allowing us to identify long-term trends in the number of studies published for each category.

The SMA analysis revealed that “*Event Matching by Training and Predicting of Videos*” and “*Object Detection and Spatio–temporal Matching*” are the two categories with the most consistent growth over time, suggesting they are likely to remain at the forefront of research in the near future. These categories benefit from the increasing advancements in machine learning and computer vision, which have driven interest in predictive techniques and spatial–temporal analysis. On the other hand, “*Multi-source Event Matching*” has shown lower but consistent growth, indicating a specialized area with steady interest. The “Other Solutions” category has demonstrated sporadic activity, probably representing emerging or less commonly applied approaches that do not fit neatly into other categories. This irregularity makes it challenging to predict a clear trajectory for this category.

Overall, the trends highlighted by the SMA indicate that training-based and spatio–temporal approaches will continue to dominate the field, while “Multi-source Event Matching” may maintain a stable, niche presence. “Other Solutions” might evolve as new experimental methods are developed and refined. These patterns provide a basis for understanding the current state of research and inform future studies in complex event matching for video streams.

### 6.2. Bias Assessment

Bias assessment is crucial in systematic reviews to ensure the reliability and validity of the synthesized evidence. It helps identify any systematic errors that might have affected the outcomes or conclusions of the included studies. Reporting bias occurs when the dissemination of research findings is influenced by the nature or direction of results, leading to the selective publication of positive or favorable findings while negative or inconclusive results remain unpublished. By conducting bias assessments, we aimed to evaluate the risk of distortion in the overall evidence due to selective reporting, thus ensuring that the conclusions drawn from the review are accurate and unbiased.

**Figure 8:**
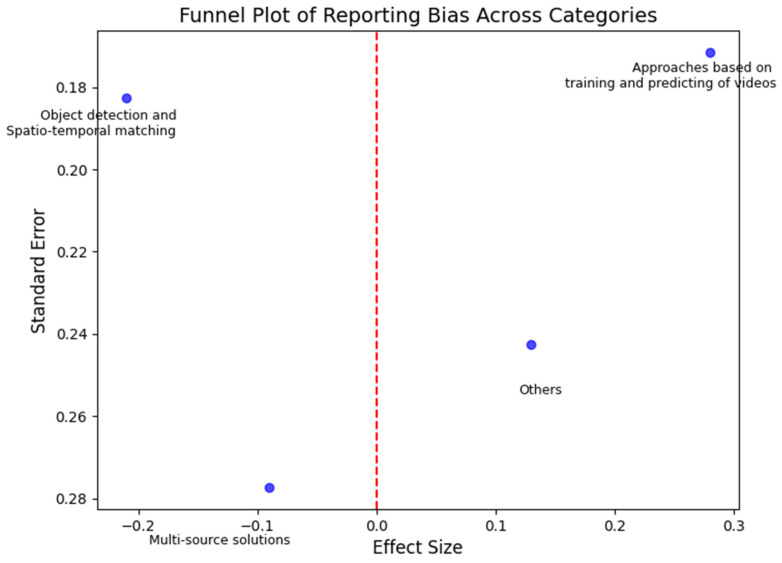
Funnel plot of the systematic review for the event-matching method categories.

Figure 8 demonstrates the symmetric distribution of data points around the central vertical line, which indicates an effect size of zero. The pattern suggests minimal reporting bias, as studies with varying sample sizes consistently report findings around a similar range of effect sizes. Larger studies with smaller standard errors are clustered towards the top, while smaller studies with larger standard errors widen towards the bottom. The absence of asymmetry indicates that both positive and negative results have been reported without selective publication, reinforcing the reliability of the findings across different categories.

### 6.3. Certainty Assessment

Certainty assessment evaluates the confidence we have in the evidence across different categories in a systematic review. For the certainty assessment, we downgraded the categories based on five factors:

- **Risk of Bias:** The level of bias in individual studies can reduce certainty. If the studies included in a category show high risk of bias (due to flaws in study design, selective reporting, or conflicts of interest), certainty is downgraded.
- **Inconsistency:** If the results across studies within a category are highly variable or inconsistent (e.g., differing effect sizes), the certainty is reduced. Consistency indicates reliable evidence, while large differences in outcomes suggest uncertainty.
- **Indirectness:** Certainty decreases if the studies do not directly address the specific research question or population. For instance, studies that only indirectly address complex event matching in videos would lower certainty.
- **Imprecision:** If studies have wide confidence intervals or include small sample sizes, they provide less precise estimates of the effect, leading to lower certainty.
- **Publication Bias:** Evidence certainty is reduced if there is suspected or confirmed reporting bias (i.e., studies with negative or non-significant findings are missing). Funnel plots and other tools help assess this bias.

The certainty of categories for this review—object detection and spatio–temporal matching, approaches based on training and predicting of videos, others, and multi-source solutions—were assessed based on these five factors. Table 6 indicates the level of certainty of the evidence for the different categories in the systematic review of complex event-matching methods for videos. Most of the studies in object detection and spatio–temporal matching (28 studies) and approaches based on training and predicting of videos (34 studies) presented consistent results with low risk of bias, giving high confidence in the findings. Smaller categories like others (17 studies) and multi-source solutions (13 studies) had slightly lower certainty due to the limited numbers of studies increasing the risk of imprecision and variability. However, the review generally maintains moderate to high certainty across categories, with larger categories providing more solid evidence, while the smaller ones need further research to strengthen confidence. The absence of major reporting biases (as shown in the bias assessment) supports this level of certainty, making the conclusions drawn from the review quite reliable.

### 6.4. Comparative Analysis of Methods by Application Domains

To provide a more comprehensive understanding of the effectiveness of the reviewed methods, we have expanded Section 6 to include a comparative analysis by application domain. This section is designed to guide researchers in choosing the most appropriate methods based on the requirements of specific domains, using a ranking system informed by the composite metric discussed earlier. Different application domains of complex event matching—such as security surveillance, smart city analytics, and sports event analysis—require varying degrees of accuracy, latency, scalability, and robustness. Below, we evaluate the suitability of methods for each domain, providing a ranking based on the composite metric.

- **Security Surveillance**

The *Event Knowledge Graph* and *Dividing Objects (Object Detection and Spatio–temporal Matching)* methods rank highly due to their accuracy and robustness in tracking dynamic and static objects. *IoT Framework (Multi-source Solutions)* also performs well due to its ability to integrate multiple data sources, which enhances robustness and detection accuracy.

- **Smart City Analytics**

*Multimodal Feature Fusion (Multi-source Solutions)* ranks highly for its balance between accuracy and scalability, making it suitable for smart city applications where multiple data types are fused. *Event Query Optimization (Object Detection and Spatio–temporal Matching)* ranks well in this domain for its capability to efficiently handle large data queries with moderate scalability.

- **Sports Event Analysis**

*Merging Frames and Vector Encoders (Event Matching by Training and Predicting)* ranks highly due to its strong performance in real-time event detection and robustness in capturing spatial–temporal relationships. The *Zero Examplers and NLP-Aided Feature Extraction (Other Solutions)* method ranks well in this domain due to its ease of implementation and flexibility in handling sports data, particularly in post-game analyses.

The comparative analysis and rankings provide practical implications for researchers:

- **New Researchers**

By using these rankings, newcomers can identify which methods are most effective in addressing the challenges relevant to their application domain.

- **Focused Development**

The rankings also highlight areas where certain methods excel or lag behind, suggesting targeted areas for improvement in future research.

By expanding Section 6 in this way, we provide a clearer comparative analysis, assisting researchers in understanding the domain-specific suitability of different methods, thereby making more informed choices in developing or enhancing complex event detection systems.

### 6.5. Categories Correlation Analysis

To assess the distinctiveness and overlap between the four categories—object detection and spatio–temporal matching, approaches based on training and predicting of videos, multi-source solutions, and others—we generated a correlation matrix (Table 7) to illustrate methodological intersections. This analysis showed that multi-source solutions maintains a limited overlap with other categories, with only two overlapping studies each with object detection and spatio-temporal matching and approaches based on training and predicting. This reflects its unique focus on integrating data from multiple sources, largely distinct from other approaches.

Similarly, the matrix indicates a small overlap (six studies) between approaches based on training and predicting and object detection and spatio–temporal matching, where both categories utilize neural networks in complementary but distinct ways—one for prediction accuracy, the other for spatial–temporal relationships. The others category exhibits minimal overlap, with only a few studies (4) shared with approaches based on training and predicting, further supporting the integrity of our categorization criteria.

Overall, this matrix demonstrates the robustness of our categories by highlighting their distinct focus areas, while identifying limited intersections where methodologies such as neural networks span multiple approaches. These findings underscore the validity of our categorizations and provide clarity on potential areas for future refinement.

## 7. Conclusions

In this paper, we have explored event-matching methods for CED in videos, covering developments over the past 12 years. These methods have been categorized into four groups based on the approaches utilized to match detected events with predefined patterns. The frequency of each category was analyzed over time and in aggregate to assess the popularity of different methods. Additionally, potential challenges in event matching identified from the studies are outlined in a separate section.

In conclusion, most of the event matching methods are based on training video CED models to predict videos. These methods are more useful for searching videos on websites often requiring semantic analysis of both videos and their captions or descriptions. However, leveraging additional data sources is the least favored approach for event matching. This method is particularly relevant for CED in live camera feeds. Nevertheless, both methods face challenges with incomplete object trajectories across consecutive frames. Due to the substantial increase in the use of closed-circuit television (CCTV) and live camera feeds for monitoring various activities, we anticipate a strong demand for CED in live camera streams across diverse applications, including industrial production, safety control, education, and exercise. Significant growth in IoT and smart devices has enabled CED to process complex events using multiple sources. Therefore, the integration of IoT and camera data to address issues with incomplete trajectories using spatio–temporal event matching would be a potential research field for the future.

## Figures and Tables

**Table 1:** Summary of research method items.

Research Method Items	Assessment Approach
Eligibility Criteria	Inclusion of 2012 to 2024, written in English, and providing quantitative results related papers
Information Sources	IEEE, Google Scholar
Search Strategy	Boolean and proximity search of keywords
Selection Process	Abstract and title screening by single reviewer
Data items	Accuracy of event matching, computational efficiency, and applicability to real-time analysis
Study Risk of Bias Assessment	Quantifying risks of reporting outcomes, selection, data input, and evaluation metrics biases by low, medium, and high scores
Synthesis Methods	Grouping the studies based on the type of event detection method and statistical and comparative analysis
Certainty Assessment	GRADE framework

**Table 2:** Summary of famous CED frameworks.

CED Framework	Processing Method
FlinkCEP	Powerful parallel processing to resolve expensive computer cluster solutions
Esper	Upside-down traditional relational database
Siddhi	Microservices and event query API for streaming
KafkaStreamsCEP	Handling state with local RocksDB stores and stateful conditions for pattern matching
Eagle	Uses a distributed stream processing framework and dynamic rule-based detection
RivusCEP	Pattern matching based on a directed-graph finite state machine and streaming data using a declarative SQL-like engine
GoStream	Running event aggregation, temporal constraints, and deriving insights from data streams.

**Table 3:** Summary of complex event-matching methods’ advantages and disadvantages.

Event-Matching Method	Advantages	Disadvantages
Multi-source solutions	- Increasing accuracy and cross-validating results; - Reducing the effect of videos quality issues; - Suitable for integrated surveillance systems.	- Low robustness due to various sources of noise; - Low scalability due to the dependency on other data sources.
Training and predicting of videos	- High accuracy due to customizing events matching prediction model.	- Low availability of training labels; - Low scalability due to the dependency on the data domain.
Object detection and Spatio-temporal matching	- High scalability due to the independence of the application domain; - High accuracy because of removing video-level error impact.	- Latency issue because of a high level of complexity for event matching; - Tracking loss of moving objects.
Other solutions	- Independence from labeled data size.	- Dependence on textual metadata for videos.

**Table 4:** Intra-category rankings for event-matching methods.

Inter-Category	Intra-Category	Accuracy	Robustness	LowLatency	Scalability	Ease of Implementation	Composite Rank
Multi-source solutions	IoT framework	high	high	moderate	high	low	high
Multi-source solutions	Multimodal feature fusion	high	moderate	moderate	low	moderate	moderate
Multi-source solutions	Low level feature integration and hierarchical event fusion	high	low	moderate	moderate	Low	low
Training and predicting of videos	Multi-level collaborative regression	moderate	moderate	high	low	moderate	moderate
Training and predicting of videos	Multiple instances learning	high	moderate	moderate	low	moderate	moderate
Training and predicting of videos	Spatial and temporal key evidence	high	moderate	moderate	moderate	moderate	moderate
Training and predicting of videos	Cell-by-cell basis feature fusion	high	high	low	low	low	low
Training and predicting of videos	Merging frames and vector encoders	high	moderate	high	low	moderate	high
Object detection and spatio–temporal matching	Abnormal behavior detection	high	moderate	moderate	low	low	moderate
Object detection and spatio–temporal matching	Event query optimization	moderate	low	high	high	moderate	moderate
Object detection and spatio–temporal matching	Event Knowledge Graph and dividing objects to static and dynamic categories	high	high	moderate	high	moderate	high
Other solutions	Feature extraction and BOW descriptors	moderate	moderate	moderate	moderate	moderate	moderate
Other solutions	Zero examplers and NLP-aided feature extraction	moderate	high	moderate	high	moderate	high

**Table 5:** Summary of Event-matching challenges and Top-ranked solutions.

Challenge	Event Matching Method	Top Rank Intra-Category Solutions
Object Detection and Tracking	Object Detection and Spatio–temporal Matching	Event Knowledge Graph and IoT Framework
Small Training Dataset Size for Visual Examples	Event Matching by Training and Predicting	Zero-Shot techniques
Optical Flow Failure	Object Detection and Spatio–temporal Matching	Event Knowledge Graph and IoT Framework
Noisy and Unreliable Labels	Event Matching by Training and Predicting and Other Solutions	Multiple Instances Learning
Latency Problem of Event Recognition	Multi-source Solutions	Serverless IoT framework
Designing Ontology of Objects and Relationships for Complex Events	Object Detection and Spatio–temporal Matching	Event Knowledge Graph and IoT Framework
Spatial Relationships Extraction	Object Detection and Spatio–temporal Matching	Event Knowledge Graph and IoT Framework

**Table 6:** Certainty of evidence for event-matching categories.

Category	Number of Studies	Risk of Bias	Inconsistency	Indirectness	Imprecision	Publication Bias	Overall Certainty
Object detection and spatio–temporal matching	30	Low	Low	Low	Low	Low	High
Approaches based on training and predicting of videos	34	Low	Low	Low	Low	Low	High
Others	17	Moderate	Moderate	Low	Moderate	Low	Moderate
Multi-source solutions	13	High	Moderate	Moderate	High	Moderate	Low

**Table 7:** Coefficient matrix for categories correlation analysis.

Category	Object Detection and Spatio–Temporal Matching	Approaches Based on Training and Predicting	Others	Multi-Source Solutions
Object Detection and Spatio–temporal Matching	30	6	3	2
Approaches Based on Training and Predicting	6	34	4	2
Others	3	4	17	1
Multi-source Solutions	2	2	1	13

## Abbreviations

**Abbreviations**	**Definitions**
CED	Complex event detection
DSMS	Data stream management system
MRFR	Market Research Future
CEP	Complex event processing
PRISMA	Preferred Reporting Items for Systematic Reviews and Meta-Analyses
GRADE	Grading of Recommendations Assessment, Development and Evaluation
CER	Complex event recognition
IoT	Internet of Things
EPL	Event Processing Language
SQL	Structured Query Language
BOW	Bag-of-Words
MAFnet	Multimodal attentive fusion network
FiLM	Feature-wise linear modulation
NLP	Natural language processing
AVRL	Audio–video representation learning
CNN	Convolutional neural network
MCR	Multi-level collaborative regression
MIL	Multiple instances learning
IDT	Improved dense trajectories
DCNN	Deep convolutional neural network
S2L	Single-StreamLine
LSTM	Long short-term memory
VEQL	Video Event Query Language
VEKG	Video Event Knowledge Graph
MERN	Multimedia event relation network
TAG	Time-aggregated graph
NOP	Notification- Oriented Paradigm
SQC	Static query chain
DQC	Dynamic query chain
SIN	Semantic Index dataset
EACI	Event-adaptive concept integration
ASUC	Area score under curve
CCTV	Closed-circuit television
SVM	Support vector machine
SIFT	Scale-invariant feature transform
GITS	Gini-Index Text
MoSIFT	Motion SIFT

## References

[1] Liang S.H., Saeedi S., Ojagh S., Honarparvar S., Kiaei S., Jahromi M.M., Squires J. (2021). An Interoperable Architecture for the Internet of COVID-19 Things (IoCT) Using Open Geospatial Standards—Case Study: Workplace Reopening. Sensors.

[2] Luckham D. (2002). The Power of Events.

[3] Yao W., Chu C.-H., Li Z. (2011). Leveraging complex event processing for smart hospitals using RFID. J. Netw. Comput. Appl..

[4] Wu E., Diao Y., Rizvi S. High-performance complex event processing over streams. Proceedings of the 2006 ACM SIGMOD International Conference on Management of Data.

[5] Wang D. (2013). Extending Complex Event Processing for Advanced Applications.

[6] Cugola G., Margara A. (2012). Processing flows of information: From data stream to complex event processing. ACM Comput. Surv. (CSUR).

[7] Coppola J. (2021). Announcing the 2021 State of Video Report.

[8] MRFR (Market Research Future) (2021). AI Camera Market Research Report: By Type (Smartphone Cameras, Surveillance Cameras, DSLRs, others), By Technology (Image/Face Recognition, Speech/Voice Recognition, Computer Vision, others) and by Region (North America, Europe, Asia-Pacific, Middle East & Africa and South America)—Forecast till 2027.

[9] Bazhenov N., Korzun D. Event-driven video services for monitoring in edge-centric internet of things environments. Proceedings of the 2019 25th Conference of Open Innovations Association (FRUCT).

[10] Vu V.-T., Brémond F., Davini G., Thonnat M., Pham Q.-C., Allezard N., Sayd P., Rouas J.-L., Ambellouis S., Flancquart A. Audio-video event recognition system for public transport security. Proceedings of the 2006 IET Conference on Crime and Security.

[11] Knoch S., Ponpathirkoottam S., Schwartz T. Video-to-model: Unsupervised trace extraction from videos for process discovery and conformance checking in manual assembly. Proceedings of the Business Process Management: 18th International Conference, BPM 2020.

[12] Li Z., Katsifodimos A., Bozzon A., Houben G. Complex Event Processing on Real-time Video Streams. Proceedings of the VLDB 2020 PhD Workshop.

[13] Yadav P., Curry E. VidCEP: Complex Event Processing Framework to Detect Spatiotemporal Patterns in Video Streams. Proceedings of the 2019 IEEE International Conference on Big Data (Big Data).

[14] Bhattacharya S., Yu F.X., Chang S.-F. Minimally needed evidence for complex event recognition in unconstrained videos. Proceedings of the International Conference on Multimedia Retrieval.

[15] Giatrakos N., Alevizos E., Artikis A., Deligiannakis A., Garofalakis M. (2020). Complex event recognition in the big data era: A survey. VLDB J..

[16] Vandenhouten R., Holland-Moritz R. (2012). A software architecture for intelligent facility management based on complex event processing. Wiss. Beiträge.

[17] Chuanfei X., Shukuan L., Lei W., Jianzhong Q. Complex event detection in probabilistic stream. Proceedings of the 2010 12th International Asia-Pacific Web Conference.

[18] Wang J., Ji B., Lin F., Lu S., Lan Y., Cheng L. (2020). A multiple pattern complex event detection scheme based on decomposition and merge sharing for massive event streams. Int. J. Distrib. Sens. Netw..

[19] Artikis A., Sergot M., Paliouras G. (2014). An event calculus for event recognition. IEEE Trans. Knowl. Data Eng..

[20] Sokha Y., Jeong K., Lee J., Joe W. (2013). A complex event processing system approach to real time road traffic event detection. J. Converg. Inf. Technol..

[21] Wang W., Sung J., Kim D. Complex event processing in epc sensor network middleware for both rfid and wsn. Proceedings of the 2008 11th IEEE International Symposium on Object and Component-Oriented Real-Time Distributed Computing (ISORC).

[22] Keskisärkkä R., Blomqvist E. Semantic complex event processing for social media monitoring-a survey. Proceedings of the Social Media and Linked Data for Emergency Response (SMILE) Co-located with the 10th Extended Semantic Web Conference.

[23] Megargel A., Shankararaman V., Reddy S.K. (2018). Real-time inbound marketing: A use case for digital banking. Handbook of Blockchain, Digital Finance, and Inclusion, Volume 1.

[24] Michelson B.M. (2006). Event-driven architecture overview. Patricia Seybold Group.

[25] Suntinger M., Obweger H., Schiefer J., Groller M.E. The event tunnel: Interactive visualization of complex event streams for business process pattern analysis. Proceedings of the 2008 IEEE Pacific Visualization Symposium.

[26] Chang X., Yu Y.-L., Yang Y., Xing E.P. (2016). Semantic pooling for complex event analysis in untrimmed videos. IEEE Trans. Pattern Anal. Mach. Intell..

[27] Khazael B., Malazi H.T., Clarke S. (2021). Complex event processing in smart city monitoring applications. IEEE Access.

[28] Dhillon A.S., Majumdar S., St-Hilaire M., El-Haraki A. A mobile complex event processing system for remote patient monitoring. Proceedings of the 2018 IEEE International Congress on Internet of Things (ICIOT).

[29] Widder A., von Ammon R., Schaeffer P., Wolff C. Combining discriminant analysis and neural networks for fraud detection on the base of complex event processing. Proceedings of the Second International Conference on Distributed Event-Based Systems.

[30] Fülöp L.J., Beszédes Á., Tóth G., Demeter H., Vidács L., Farkas L. Predictive complex event processing: A conceptual framework for combining complex event processing and predictive analytics. Proceedings of the Fifth Balkan Conference in Informatics.

[31] Liu X., Cao J., Tang S., Guo P. (2015). Fault tolerant complex event detection in WSNs: A case study in structural health monitoring. IEEE Trans. Mob. Comput..

[32] Naseri M.M., Tabibian S., Homayounvala E. (2022). Adaptive and personalized user behavior modeling in complex event processing platforms for remote health monitoring systems. Artif. Intell. Med..

[33] Wang D., Rundensteiner E.A., Wang H., Ellison III R.T. (2010). Active complex event processing: Applications in real-time health care. Proc. VLDB Endow..

[34] Honarparvar S., Saeedi S., Liang S., Squires J. (2021). Design and Development of an Internet of Smart Cameras Solution for Complex Event Detection in COVID-19 Risk Behaviour Recognition. ISPRS Int. J. Geo-Inf..

[35] de Moura I.R., e Silva F.J.d.S., Coutinho L.R., Teles A.S. Mental health ubiquitous monitoring: Detecting context-enriched sociability patterns through complex event processing. Proceedings of the 2020 IEEE 33rd International Symposium on Computer-Based Medical Systems (CBMS).

[36] Wang Y., Cao K. (2014). A proactive complex event processing method for large-scale transportation internet of things. Int. J. Distrib. Sens. Netw..

[37] Wang Y., Cao K., Zhang X. (2013). Complex event processing over distributed probabilistic event streams. Comput. Math. Appl..

[38] Hussonnois F. Kafka Streams CEP. https://github.com/fhussonnois/kafkastreams-cep.

[39] Lu J. Eagle: Real Time Data Processing System Based on Flink and CEP. https://github.com/luxiaoxun/eagle.

[40] Kolarov V., Sedlacek J., Badger T.G. Rivus CEP. https://github.com/vascokk/rivus_cep.

[41] Itsubaki Gostream. https://github.com/itsubaki/gostream.

[42] Kougioumtzi E., Kontaxakis A., Deligiannakis A., Kotidis Y. Towards creating a generalized complex event processing operator using FlinkCEP: Architecture & benchmark. Proceedings of the 15th ACM International Conference on Distributed and Event-Based Systems.

[43] Bernhardt T., Kaicao, Choly I., Yang G., Shelton M., Leitschuh J. Esper—Complex Event Processing, Streaming SQL and Event Series Analysis for Java. https://github.com/espertechinc/esper.

[44] Alevizos E., Artikis A. Being logical or going with the flow? A comparison of complex event processing systems. Proceedings of the Hellenic Conference on Artificial Intelligence.

[45] Albek E., Bax E., Billock G., Chandy K.M., Swett I. An event processing language (epl) for building sense and respond applications. Proceedings of the 19th IEEE International Parallel and Distributed Processing Symposium.

[46] Suhothayan S., Gajasinghe K., Loku Narangoda I., Chaturanga S., Perera S., Nanayakkara V. Siddhi: A second look at complex event processing architectures. Proceedings of the 2011 ACM Workshop on Gateway Computing Environments.

[47] Cardinale Y., Freites G., Valderrama E., Aguilera A., Angsuchotmetee C. (2022). Semantic framework of event detection in emergency situations for smart buildings. Digit. Commun. Netw..

[48] Barbieri D.F., Braga D., Ceri S., Della Valle E., Grossniklaus M. C-SPARQL: SPARQL for continuous querying. Proceedings of the 18th International Conference on World Wide Web.

[49] Anicic D., Fodor P., Rudolph S., Stojanovic N. EP-SPARQL: A unified language for event processing and stream reasoning. Proceedings of the 20th International Conference on World Wide Web.

[50] Hasan S., Curry E. (2014). Approximate semantic matching of events for the internet of things. ACM Trans. Internet Technol. (TOIT).

[51] Teymourian K., Coskun G., Paschke A. (2010). Modular Upper-Level Ontologies for Semantic Complex Event Processing. Modular Ontologies.

[52] Binnewies S., Stantic B. OECEP: Enriching complex event processing with domain knowledge from ontologies. Proceedings of the Fifth Balkan Conference in Informatics.

[53] Doulamis N.D., Kokkinos P., Varvarigos E. (2012). Resource selection for tasks with time requirements using spectral clustering. IEEE Trans. Comput..

[54] Akdere M., Çetintemel U., Tatbul N. (2008). Plan-based complex event detection across distributed sources. Proc. VLDB Endow..

[55] Jones M.T. (2013). Process Real-Time Big Data with Twitter Storm.

[56] Zhou Q., Simmhan Y., Prasanna V. (2017). Knowledge-infused and consistent Complex Event Processing over real-time and persistent streams. Future Gener. Comput. Syst..

[57] Esposito C., Ficco M., Palmieri F., Castiglione A. (2015). A knowledge-based platform for big data analytics based on publish/subscribe services and stream processing. Knowl.-Based Syst..

[58] Skarlatidis A., Paliouras G., Artikis A., Vouros G.A. (2015). Probabilistic event calculus for event recognition. ACM Trans. Comput. Log. (TOCL).

[59] Liu F., Deng D., Li P. (2017). Dynamic context-aware event recognition based on Markov logic networks. Sensors.

[60] Rincé R., Kervarc R., Leray P. Complex event processing under uncertainty using Markov chains, constraints, and sampling. Proceedings of the International Joint Conference on Rules and Reasoning.

[61] Wang J., Cheng L., Liu J. (2014). A Complex Event Detection Method formulti-probability RFID Event Stream. J. Softw..

[62] Cugola G., Margara A., Matteucci M., Tamburrelli G. (2015). Introducing uncertainty in complex event processing: Model, implementation, and validation. Computing.

[63] Natarajan P., Wu S., Vitaladevuni S., Zhuang X., Tsakalidis S., Park U., Prasad R., Natarajan P. Multimodal feature fusion for robust event detection in web videos. Proceedings of the 2012 IEEE Conference on Computer Vision and Pattern Recognition.

[64] Jhuo I.-H., Lee D. Video event detection via multi-modality deep learning. Proceedings of the 2014 22nd International Conference on Pattern Recognition.

[65] Oh S., McCloskey S., Kim I., Vahdat A., Cannons K.J., Hajimirsadeghi H., Mori G., Perera A.A., Pandey M., Corso J.J. (2014). Multimedia event detection with multimodal feature fusion and temporal concept localization. Mach. Vis. Appl..

[66] Shahad R.A., Bein L.G., Saad M.H.M., Hussain A. Complex event detection in an intelligent surveillance system using CAISER platform. Proceedings of the 2016 International Conference on Advances in Electrical, Electronic and Systems Engineering (ICAEES).

[67] Brousmiche M., Rouat J., Dupont S. (2022). Multimodal Attentive Fusion Network for audio-visual event recognition. Inf. Fusion.

[68] Gao J., Yang H., Gong M., Li X. (2024). Audio–visual representation learning for anomaly events detection in crowds. Neurocomputing.

[69] Ma Z., Yang Y., Xu Z., Yan S., Sebe N., Hauptmann A.G. Complex event detection via multi-source video attributes. Proceedings of the IEEE Conference on Computer Vision and Pattern Recognition.

[70] Tang K., Fei-Fei L., Koller D. Learning latent temporal structure for complex event detection. Proceedings of the 2012 IEEE Conference on Computer Vision and Pattern Recognition.

[71] Yang Y., Ma Z., Xu Z., Yan S., Hauptmann A.G. How related exemplars help complex event detection in web videos?. Proceedings of the IEEE International Conference on Computer Vision.

[72] Lai K.-T., Yu F.X., Chen M.-S., Chang S.-F. Video event detection by inferring temporal instance labels. Proceedings of the IEEE Conference on Computer Vision and Pattern Recognition.

[73] Xu Z., Tsang I.W., Yang Y., Ma Z., Hauptmann A.G. Event detection using multi-level relevance labels and multiple features. Proceedings of the IEEE Conference on Computer Vision and Pattern Recognition.

[74] Gan C., Wang N., Yang Y., Yeung D.-Y., Hauptmann A.G. Devnet: A deep event network for multimedia event detection and evidence recounting. Proceedings of the IEEE Conference on Computer Vision and Pattern Recognition.

[75] Abbasnejad I., Sridharan S., Denman S., Fookes C., Lucey S. Complex event detection using joint max margin and semantic features. Proceedings of the 2016 International Conference on Digital Image Computing: Techniques and Applications (DICTA).

[76] Liu H., Zheng Q., Li Z., Qin T., Zhu L. (2018). An efficient multi-feature SVM solver for complex event detection. Multimed. Tools Appl..

[77] Dao M.-S., Zettsu K. Complex event analysis of urban environmental data based on deep CNN of spatiotemporal raster images. Proceedings of the 2018 IEEE International Conference on Big Data (Big Data).

[78] Xu Z., Su L., Wang S., Huang Q., Zhang Y. S2L: Single-streamline for complex video event detection. Proceedings of the 2018 IEEE International Conference on Multimedia & Expo Workshops (ICMEW).

[79] Luo M., Chang X., Gong C. (2021). Reliable shot identification for complex event detection via visual-semantic embedding. Comput. Vis. Image Underst..

[80] Alanazi T., Muhammad G. (2022). Human fall detection using 3D multi-stream convolutional neural networks with fusion. Diagnostics.

[81] Vilamala M.R., Xing T., Taylor H., Garcia L., Srivastava M., Kaplan L., Preece A., Kimmig A., Cerutti F. (2023). DeepProbCEP: A neuro-symbolic approach for complex event processing in adversarial settings. Expert Syst. Appl..

[82] Fei M., Jiang W., Mao W. (2023). Creating personalized video summaries via semantic event detection. J. Ambient Intell. Humaniz. Comput..

[83] Chakraborty I., Cheng H., Javed O. Entity Centric Feature Pooling for Complex Event Detection. Proceedings of the 1st ACM International Workshop on Human Centered Event Understanding from Multimedia.

[84] Ye G., Li Y., Xu H., Liu D., Chang S.-F. Eventnet: A large scale structured concept library for complex event detection in video. Proceedings of the 23rd ACM International Conference on Multimedia.

[85] Chen C.Y., Fu J.H., Sung T., Wang P.-F., Jou E., Feng M.-W. Complex event processing for the internet of things and its applications. Proceedings of the 2014 IEEE International Conference on Automation Science and Engineering (CASE).

[86] Ke J., Chen X.-J., Chen B.-D., Xu H., Zhang J.-G., Jiang X.-M., Wang M.-R., Chen X.-B., Zhang Q.-Q., Cai W.-H. Complex Event Detection in Video Streams. Proceedings of the 2016 IEEE Symposium on Service-Oriented System Engineering (SOSE).

[87] Coşar S., Donatiello G., Bogorny V., Garate C., Alvares L.O., Brémond F. (2016). Toward abnormal trajectory and event detection in video surveillance. IEEE Trans. Circuits Syst. Video Technol..

[88] Li C., Huang Z., Yang Y., Cao J., Sun X., Shen H.T. (2017). Hierarchical latent concept discovery for video event detection. IEEE Trans. Image Process..

[89] Zhang H., Ananthanarayanan G., Bodik P., Philipose M., Bahl P., Freedman M.J. Live video analytics at scale with approximation and {Delay-Tolerance}. Proceedings of the 14th USENIX Symposium on Networked Systems Design and Implementation (NSDI 17).

[90] Kang D., Bailis P., Zaharia M. (2018). BlazeIt: Optimizing declarative aggregation and limit queries for neural network-based video analytics. arXiv.

[91] Khan A., Serafini L., Bozzato L., Lazzerini B. Event Detection from Video Using Answer Set Programing. Proceedings of the CILC.

[92] Yadav P., Curry E. VEKG: Video Event Knowledge Graph to Represent Video Streams for Complex Event Pattern Matching. Proceedings of the 2019 First International Conference on Graph Computing (GC).

[93] Yadav P. High-performance complex event processing framework to detect event patterns over video streams. Proceedings of the 20th International Middleware Conference Doctoral Symposium.

[94] Yadav P., Curry E. (2020). Visual Semantic Multimedia Event Model for Complex Event Detection in Video Streams. arXiv.

[95] Yadav P., Salwala D., Das D.P., Curry E. (2020). Knowledge Graph Driven Approach to Represent Video Streams for Spatiotemporal Event Pattern Matching in Complex Event Processing. Int. J. Semant. Comput..

[96] Patel A.S., Merlino G., Puliafito A., Vyas R., Vyas O., Ojha M., Tiwari V. (2023). An NLP-guided ontology development and refinement approach to represent and query visual information. Expert Syst. Appl..

[97] Kossoski C., Simão J.M., Lopes H.S. (2024). Modeling and Performance Analysis of a Notification-Based Method for Processing Video Queries on the Fly. Appl. Sci..

[98] Tamrakar A., Ali S., Yu Q., Liu J., Javed O., Divakaran A., Cheng H., Sawhney H. Evaluation of low-level features and their combinations for complex event detection in open source videos. Proceedings of the 2012 IEEE Conference on Computer Vision and Pattern Recognition.

[99] Yan Y., Yang Y., Meng D., Liu G., Tong W., Hauptmann A.G., Sebe N. (2015). Event oriented dictionary learning for complex event detection. IEEE Trans. Image Process..

[100] Chang X., Yang Y., Long G., Zhang C., Hauptmann A. Dynamic concept composition for zero-example event detection. Proceedings of the AAAI Conference on Artificial Intelligence.

[101] de Boer M., Schutte K., Kraaij W. (2016). Knowledge based query expansion in complex multimedia event detection. Multimed. Tools Appl..

[102] Li Z., Yao L., Chang X., Zhan K., Sun J., Zhang H. (2019). Zero-shot event detection via event-adaptive concept relevance mining. Pattern Recognit..

[103] Li Z., Chang X., Yao L., Pan S., Zongyuan G., Zhang H. Grounding visual concepts for zero-shot event detection and event captioning. Proceedings of the 26th ACM SIGKDD International Conference on Knowledge Discovery & Data Mining.

[104] Jin Y., Jiang W., Yang Y., Mu Y. (2021). Zero-Shot Video Event Detection with High-Order Semantic Concept Discovery and Matching. IEEE Trans. Multimed..

[105] Mazon-Olivo B., Hernández-Rojas D., Maza-Salinas J., Pan A. (2018). Rules engine and complex event processor in the context of internet of things for precision agriculture. Comput. Electron. Agric..

[106] Mousheimish R. (2017). Combining the Internet of Things, Complex Event Processing, and Time Series Classification for a Proactive Business Process Management. Ph.D. Thesis.

